# Supplementary material for: Activating the NFE2L1-ubiquitin-proteasome system by DDI2 protects from ferroptosis
Source: Cell Death Differ. 2024 Oct 9;32(3):480–7. doi: 10.1038/s41418-024-01398-z (PMC11893739; doi:10.1038/s41418-024-01398-z)
Supplement: Supplementary file 1 — Supplementary Figures 1-5 [file 41418_2024_1398_MOESM1_ESM.pdf]

Supplementary figures

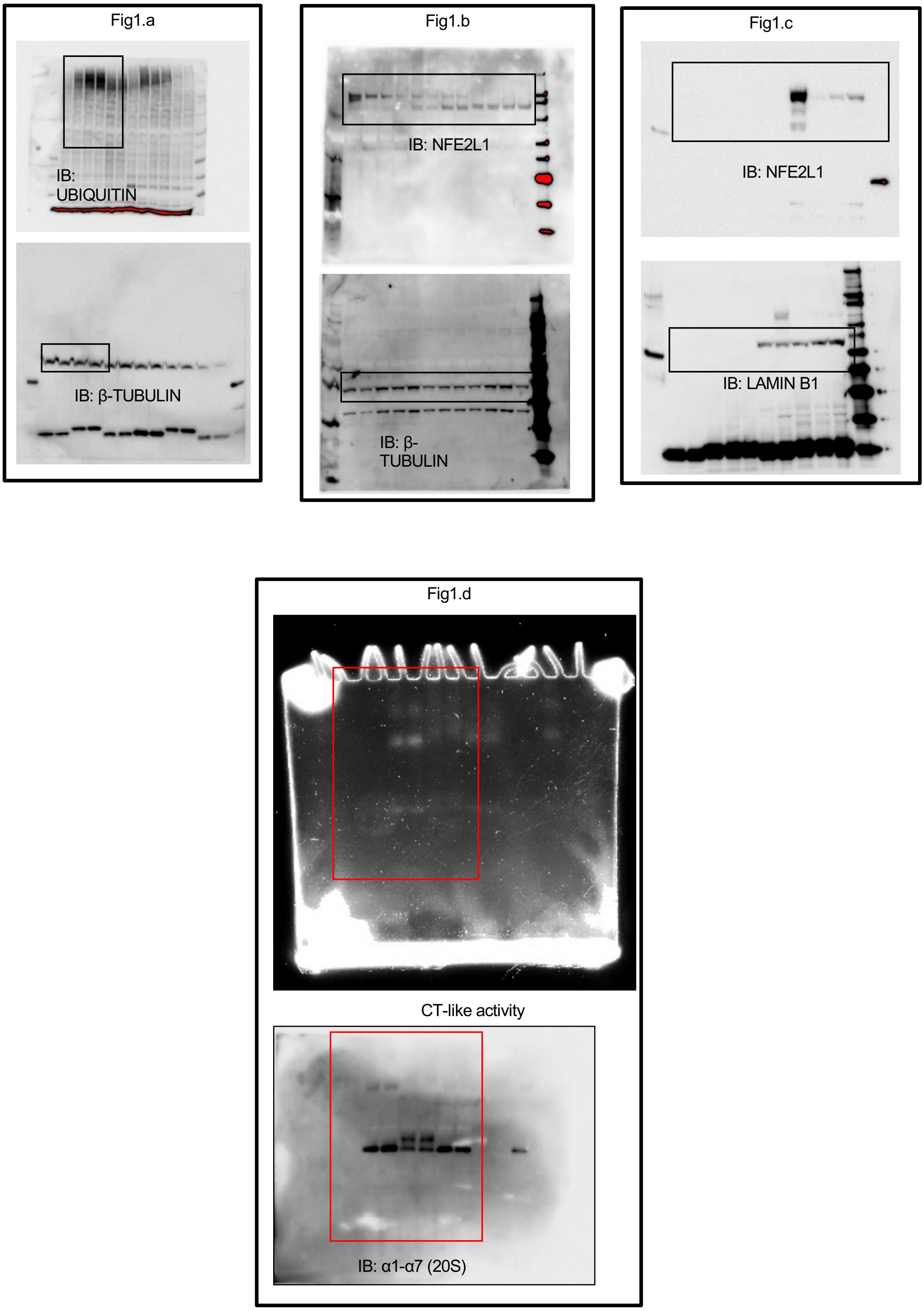

Supplementary Fig. S1: Uncropped immunoblots and gel pictures

Supplementary figures

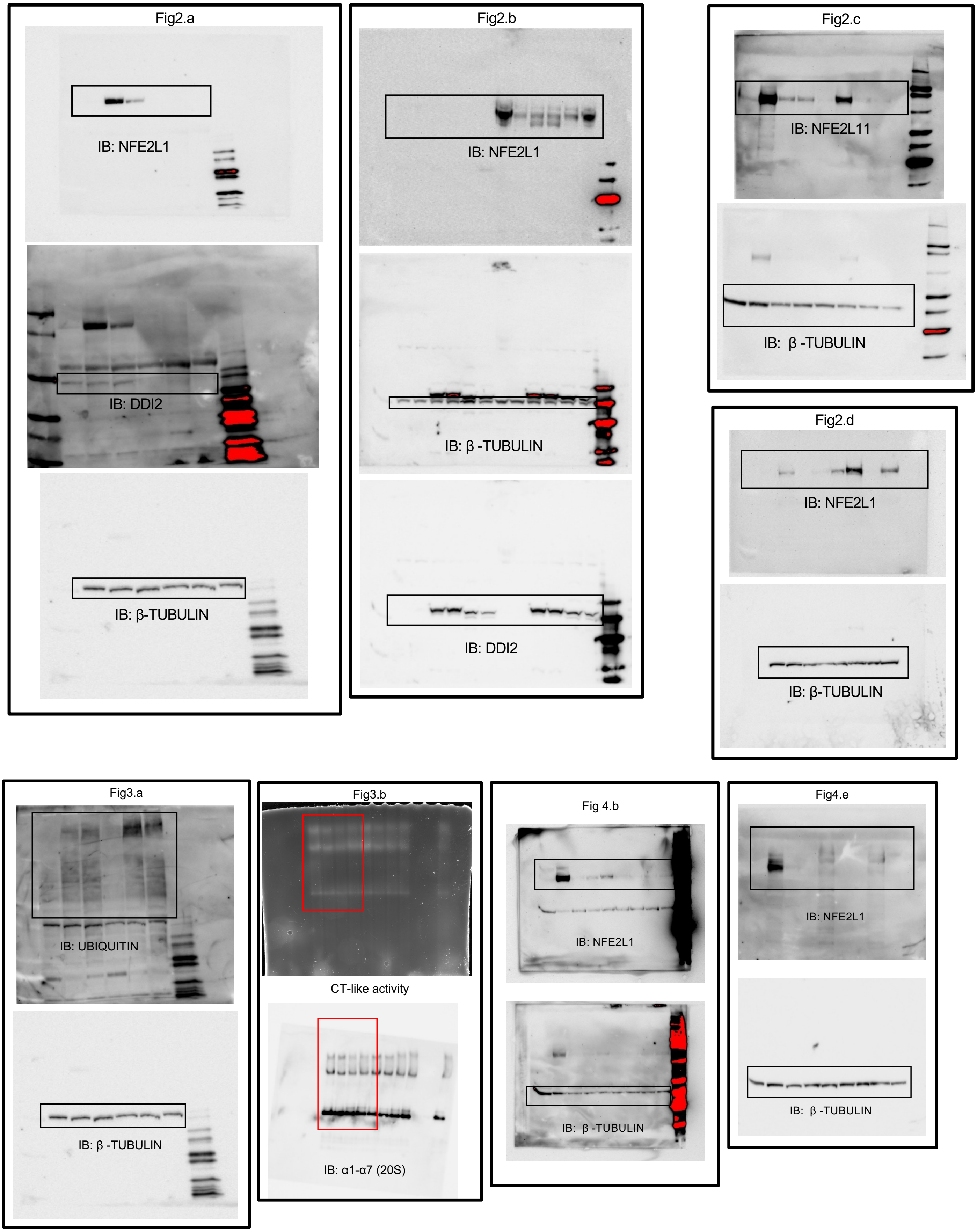

Supplementary Fig. S2: Uncropped immunoblots

Supplementary figures

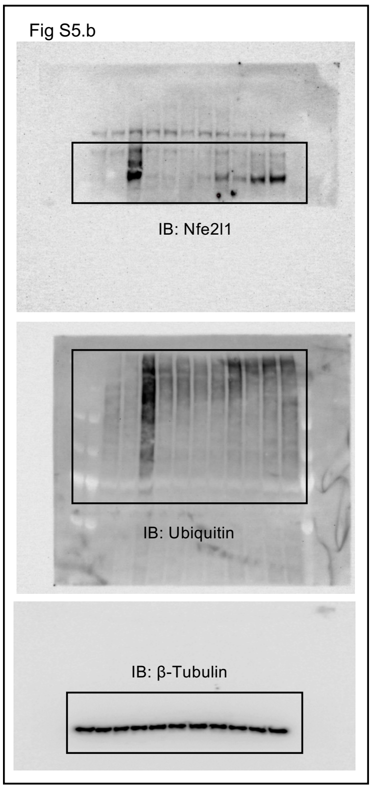

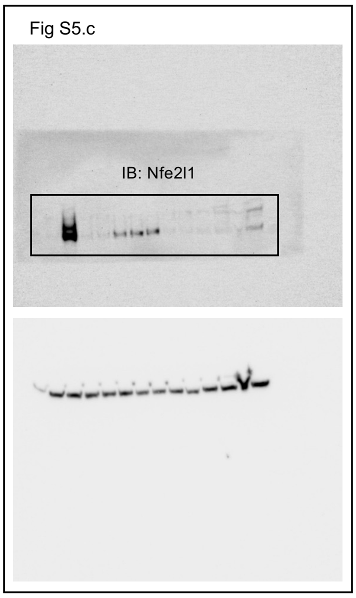

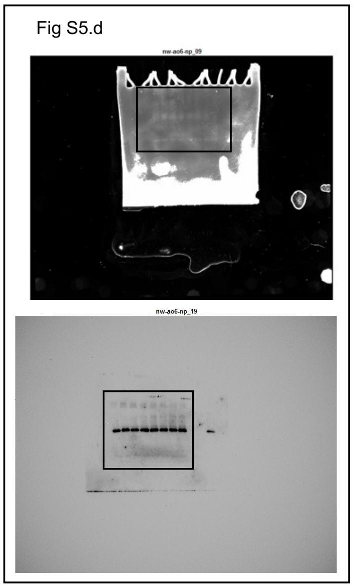

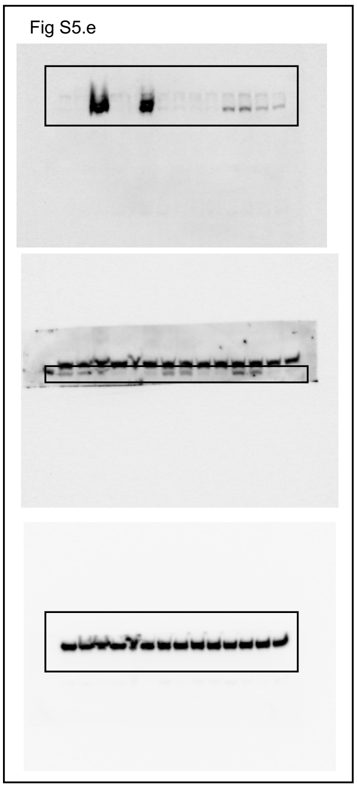

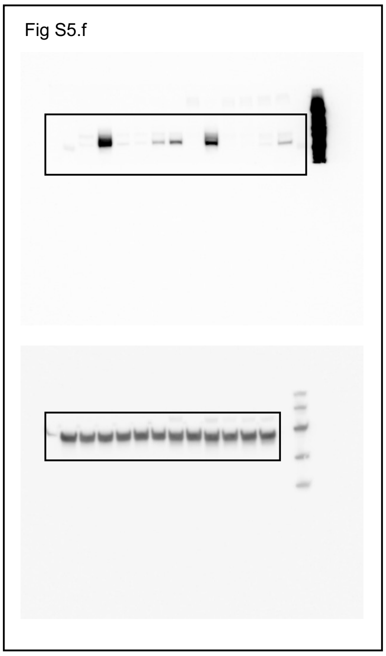

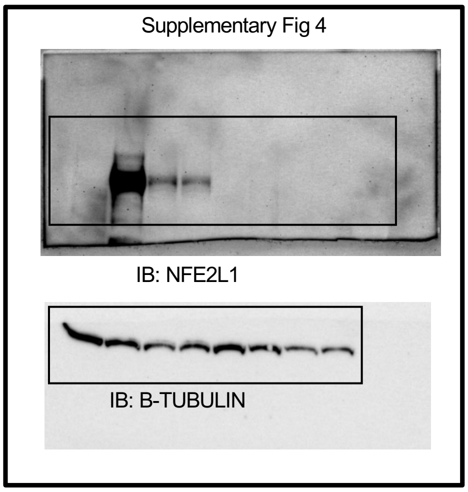

Supplementary Fig. S3: Uncropped immunoblots and gel pictures

Supplementary figures

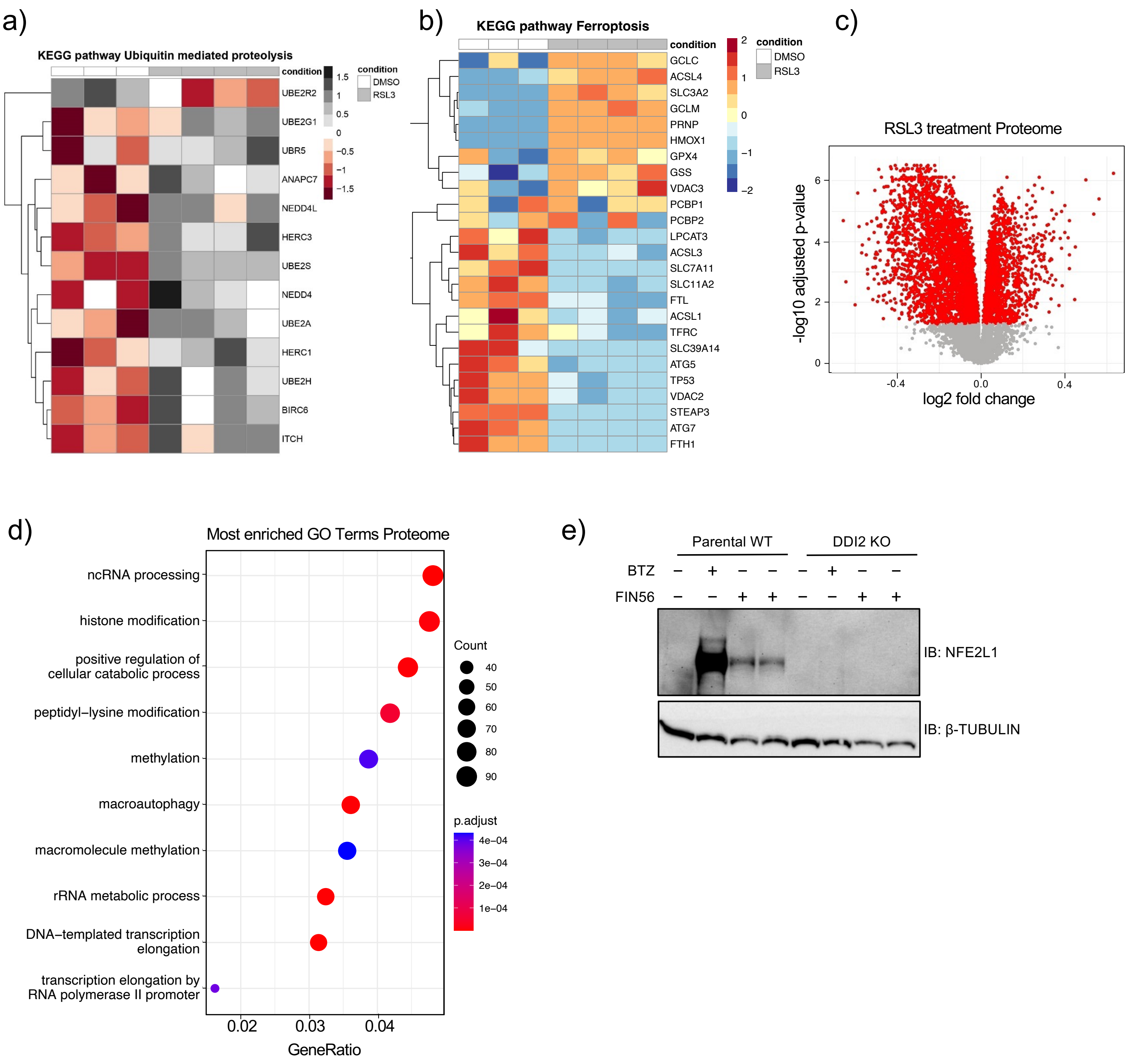

Supplementary Fig. S4.: (a) KEGG pathway analysis of ubiquitin mediated proteolysis from ubiquitome of EaHy926 cells treated with 5 µM of RSL3 in the total proteome. (b) KEGG pathway analysis of ferroptosis from total proteome of EaHy926 cells treated with 5 µM of RSL3. (c) Volcano Plot of the proteome of EaHy926 cells treated for 9 h with 5 µM RSL3 $P_{adj} < 0.05$ indicated by red color. (n=4 replicates) (d) Top 10 enriched gene ontology (GO) terms in the proteome of EaHy926 cells treated for 9 h with 5 µM RSL3. (e) Immunoblot of NFE2L1 in wild-type EA.hy926 and DDI2 KO cells treated with 10 µM FIN56 for 9 h and 100 nM BTZ for 3 h.

Supplementary figures

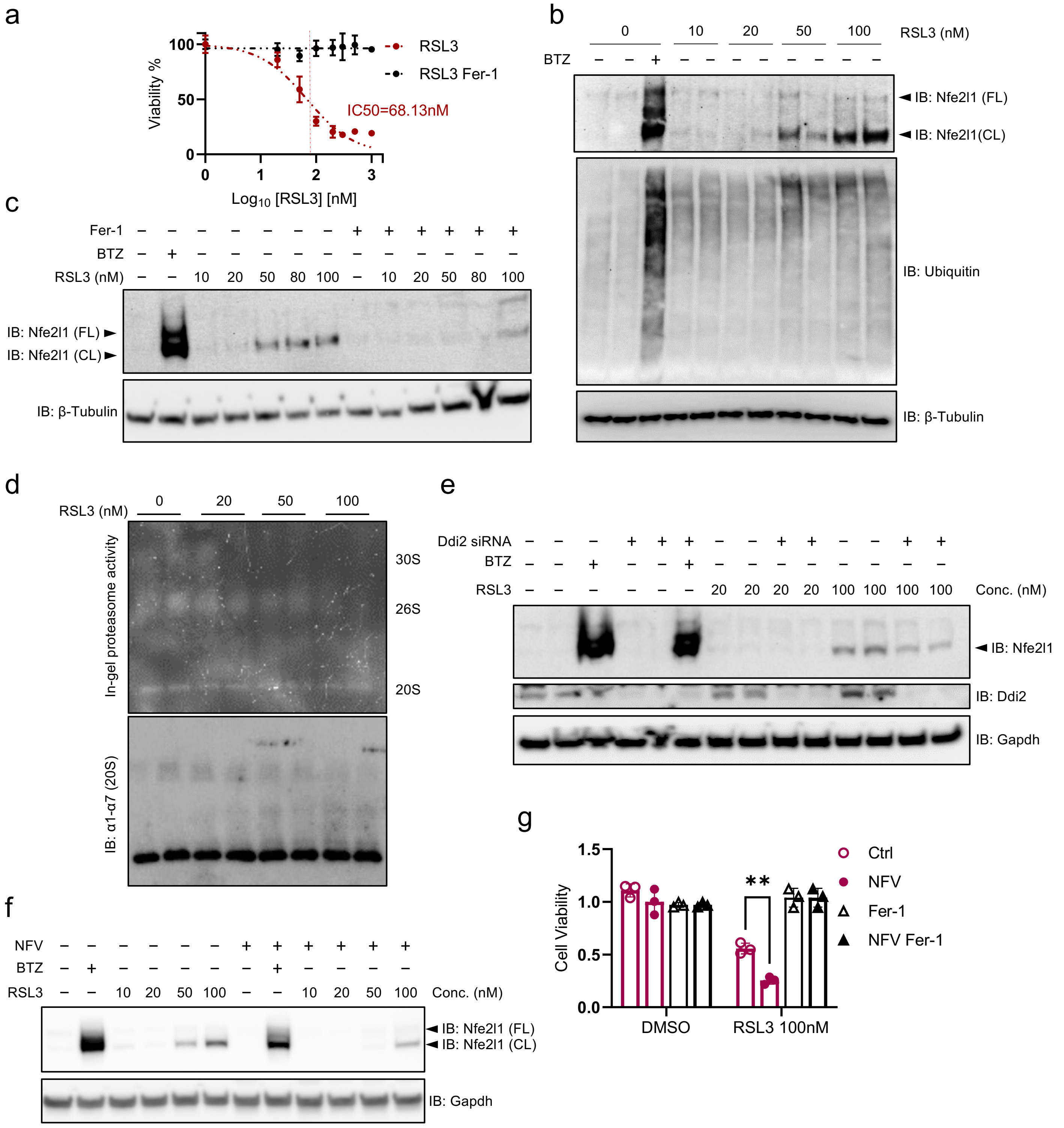

Supplementary Fig. S5: RSL3-dependent regulation of the Nfe2l1-proteasome pathway by Ddi2 in WT1 cells. (a) RSL3 dose-response curve in the presence or absence of 1 μM Ferrostatin-1 (Fer-1), cell death after 6 h measured by AquaBlueR. (b) Impact of RSL3 treatment for 6 h on Nfe2l1 and ubiquitin levels and (c) effects of co-incubation with 10 μM Fer-1. (d) Native PAGE for measuring proteasome assembly and activity. (e) Effect of silencing Ddi2 on RSL3-dependent Nfe2l1 protein levels. (f) Effect of Nelfinavir (NFV) on RSL3-dependent Nfe2l1 protein levels. (g) RSL3-dependent cell viability with co-treatments of either 10 μM NFV or 10 μM Fer-1 for 20 h. Treatment with 50 nM Bortezomib (BTZ) for 3 h served as a positive control for Nfe2l1 activation.
