# Supplementary material for: Activating the NFE2L1-ubiquitin-proteasome system by DDI2 protects from ferroptosis
Source: Cell Death Differ. 2024 Oct 9;32(3):480–7. doi: 10.1038/s41418-024-01398-z (PMC11893739; doi:10.1038/s41418-024-01398-z)
Supplement: Supplementary file 2 — Supplementary tables 1-2 [file 41418_2024_1398_MOESM2_ESM.docx]

**Supplementary Table 1: Antibody list**

| Antibody | Company (Cat. No.) | Dilution |
| --- | --- | --- |
| DDI2 | Abcam (ab197081) | 1:1000 |
| NFE2L1 | Cell signaling technologies (D5B10) | 1:500 |
| β-TUBULIN | Cell signaling technologies (2146) | 1:1000 |
| UBIQUITIN (P4D1) | Cell signaling technologies (3936) | 1:1000 |
| 20S Proteasome ɑ subunit | Abcam (ab22674) | 1:1000 |
| Anti-Lamin B1 | Abcam (ab229025) | 1:1000 |
| Anti-rabbit IgG, HRP-linked | Cell signaling technologies (7074) | 1:10000 |
| Anti-mouse IgG, HRP-linked | Cell signaling technologies (7076) | 1:10000 |

**Supplementary Table 2: Primer list**

| Gene | Forward Primer (5’) | Reverse Primer (5’) |
| --- | --- | --- |
| *NFE2L1* | AGTGGAGACTTAACCAAAGAGGAC | CTCCTTCTGGCGGTGACTAT |
| *TBP* | CCCATGACTCCCATGACC | TTTACAACCAAGATTCACTGTGG |
